# Sex Differences in Fiber Connection between the Striatum and Subcortical and Cortical Regions

**DOI:** 10.3389/fncom.2016.00100

**Published:** 2016-09-23

**Authors:** Xuemei Lei, Zhuo Han, Chuansheng Chen, Lu Bai, Gui Xue, Qi Dong

**Affiliations:** ^1^School of Psychology, Beijing Normal UniversityBeijing, China; ^2^Department of Psychology and Social Behavior, University of CaliforniaIrvine, CA, USA; ^3^National Key Laboratory of Cognitive Neuroscience and Learning, Beijing Normal UniversityBeijing, China

**Keywords:** striatum, putamen, caudate, diffusion tensor imaging, fiber connection, sex difference

## Abstract

The striatum is an important subcortical structure with extensive connections to other regions of the brain. These connections are believed to play important roles in behaviors such as reward-related processes and impulse control, which show significant sex differences. However, little is known about sex differences in the striatum-projected fiber connectivity. The current study examined sex differences between 50 Chinese males and 79 Chinese females in their fiber connections between the striatum and nine selected cortical and subcortical regions. Despite overall similarities, males showed stronger fiber connections between the left caudate and rostral cingulate cortex, between the right putamen and the lateral orbitofrontal cortex, between the bilateral putamen and the ventro-lateral prefrontal cortex, and between the right caudate and the ventro-lateral prefrontal cortex, whereas females showed stronger fiber connections between the right putamen and the dorsolateral prefrontal cortex, between bilateral caudate and hippocampus, and between the left putamen and hippocampus. These findings help us to understand sex differences in the striatum-projected fiber connections and their implications for sex differences in behaviors.

## Introduction

The striatum is an important subcortical component of the basal ganglia-thalamo-cortical circuits. The rostral, medial, and ventral parts of the striatum are primarily connected to the ventral and medial prefrontal cortex, anterior cingulate cortex, and orbitofrontal cortex, whereas the dorsal striatum, including the head of caudate and part of rostral putamen, are connected to the dorsal and lateral prefrontal cortex (Haber et al., 2006; Haber and Knutson, 2010). These fiber connections provide anatomical support for dynamic and reciprocal signaling between the striatum and other brain regions, which underlie diverse psychological functions (Pauli et al., 2016). Indeed, five distinct striatal zones have been found to be linked to distinct brain functions: the anterior caudate for incentive behaviors and the evaluation of different actions, the posterior caudate for executive functions, the posterior putamen for sensorimotor processes, the anterior putamen for social and language-related functions, and the ventral striatum for the representation of stimulus value and related stimulus-driven motivational states (Pauli et al., 2016).

Studies have reported significant sex differences in reward-related processes and impulse control, which are sub-served by the striatal-cortical and striatal-subcortical circuits (Lighthall et al., 2012; Fattore, 2015). As one example, males and females differ in processing reward information and decision making. Studies found that the putamen showed a higher reward-related anticipatory response in males than in females (Dreher et al., 2007). Compared to females, males were faster and gained greater rewards in risky decision making, and they also showed increased activation in the dorsal striatum including the putamen (Lighthall et al., 2012). Sometimes even when the behaviors showed no sex differences, brain activations differed by sex. For example, when asked to reject immediate rewards in pursuit of a long-term goal, males showed a stronger reduction of activation than did females in the dorsal striatum, subgenual and pregenual anterior cingulate cortex, posterior orbitofrontal cortex, as well as more positive functional coupling between these regions (Diekhof et al., 2012). It seems that the striatal-frontal circuits are more frequently and strongly recruited during reward-related processing in males than in females. Based on the above results, we may also speculate that males made a greater effort (as shown in more positive functional coupling) to effectively suppress the stronger response (stronger reduction of activation) that were originally activated by immediate rewards.

Although researchers have discussed extensively sex differences in the structure and function of the human brain (Cahill, 2006; Gong et al., 2009, 2011), specific sex differences within the striatum-connected structural circuits remain largely unknown mainly because of the limitations of earlier approaches to analyzing diffusion tensor imaging (DTI) data. Three approaches of analyzing DTI data have been developed: tract-based spatial statistics (TBSS), deterministic tracking, and probabilistic tractography. The TBSS method provides the most commonly used fractional anisotropy (FA) of fiber tracts, which quantifies directional strength of each voxel of the local tracts. The TBSS method does not directly quantify connections between brain regions (Smith et al., 2006). With the deterministic tracking method, seeds were placed in voxels with FA greater than a given threshold (e.g., 15) to include only white matter voxels, and then grown in both directions along the dominant diffusion orientation of voxels into fiber tracts or streamlines. Deterministic tracking has a limited capacity for resolving crossing fiber bundles, and consequently misses some fiber bundles for the lateral cortical regions (Mori and van Zijl, 2002). Probabilistic tractography builds up distributions on diffusion parameters at each voxel by using sampling techniques, and then samples from the distributions on voxel-wise principal diffusion directions, each time computing a streamline through these local samples to generate a probabilistic streamline or a sample. Probabilistic tractography is the preferred method because it is better at handling fiber crossings and image noise (Behrens et al., 2003a), although it may lead to spurious connections (Parker and Alexander, 2005). Another drawback of probabilistic tractography is that it is quite time-consuming and computationally intensive. Therefore, few studies have used this method to study specific brain circuits, and even fewer on sex differences of specific brain circuits. Consequently, even though the anatomical connection pattern of the striatum has been revealed by animal studies, postmortem human studies, and *in vivo* human brain imaging studies of small samples (Haber et al., 2006; Leh et al., 2007; Haber and Knutson, 2010), and several studies of small samples have investigated the associations between striatum-projected fiber connection and behavioral and physiological measures (Cohen et al., 2009; Bohanna et al., 2011; Lei et al., 2014; Tziortzi et al., 2014), no study has examined sex differences in the striatum-projected structural connectivity.

The current study was designed to explore sex differences in anatomical connectivity of the striatum to cortical and subcortical regions. Following previous studies (Cohen et al., 2009; Lei et al., 2014), the striatum and nine target masks (see Figure S1 for anatomical locations of these masks) for each hemisphere were created based on the automated anatomical labeling template (Tzourio-Mazoyer et al., 2002). The nine target regions included the medial orbitofrontal cortex (mOFC), lateral orbitofrontal cortex (lOFC), ventrolateral prefrontal cortex (vlPFC), dorsolateral prefrontal cortex (dlPFC), posterior cingulate cortex/retrosplenial cortex (PCC), rostral cingulate cortex (rostral CC), dorsal cingulate cortex (dorsal CC), hippocampus, and amygdala. Based on the previous studies showing that males relied on the striatal-frontal circuits to a greater extent than did females (Dreher et al., 2007; Diekhof et al., 2012; Lighthall et al., 2012), the current study hypothesized stronger fiber connection in males than in females between the striatum and the anterior cingulate cortex and orbitofrontal cortex.

## Materials and methods

### Participants

Fifty male and seventy nine female college students (mean age 20.10 years; range 19–22 years) were recruited from Beijing Normal University. All participants were right-handed Han Chinese with normal or corrected-to-normal vision. They self-reported having no history of neurological or psychiatric illnesses. They also passed the physical and clinical examinations for all freshmen administered by the University. Participants were scanned for diffusion tensor and high resolution 3D anatomical images. They all gave informed written consents and the study was approved by the Beijing Normal University Institutional Review Board.

### Image acquisition

Participants were scanned on a Siemens Trio 3T scanner with an eight-channel head coil in the Beijing Normal University Imaging Center for Brain Research. The diffusion-weighted data were acquired using a twice-refocused spin-echo EPI sequence with the following parameters: TR/TE = 7200/104 ms, 49 transverse slices, field-of-view = 230^*^230 mm, matrix = 128^*^128, slice thickness = 2.5 mm, 1 direction with *b*-value = 0 s/mm^2^, 64 directions with *b*-value = 1000 s/mm^2^. In addition, a high resolution 3D anatomical image was obtained using T1-weighted MP-RAGE sequence with the following parameters: TR/TE/FA = 2530/3.75 ms/7°, FOV = 220^*^220 mm, matrix = 256^*^256, slice thickness = 1 mm, 128 sagittal slices). Scanning lasted 18 min for each participant.

### Image preprocessing

Diffusion tensor images (DTI) were processed using the FMRIB's Diffusion Toolbox (FDT 2.0; Smith et al., 2006) from the FMRIB's Software Library (FSL, version 5.0.5; www.fmrib.ox.ac.uk/fsl; Smith et al., 2004; Woolrich et al., 2009; Jenkinson et al., 2012). The standard preprocessing procedure, including correction of the diffusion data for eddy currents and head motion, fitting of diffusion tensor, and Bayesian estimation of diffusion parameters, was used for the probabilistic tractography of DTI data (Behrens et al., 2003b, 2007; Johansen-Berg et al., 2005). Bayesian estimation of diffusion parameters was conducted with a dual-fiber model allowing for crossing fibers by using the BedpostX program implemented in FMRIB's diffusion toolbox (Behrens et al., 2003b). Detailed preprocessing steps were described in our previous study (Lei et al., 2014).

The non-diffusion-weighted images of all participants were spatially normalized into the Montreal Neurological Institute (MNI) standard space with FMRIB's Linear Image Registration Tool (FLIRT; Jenkinson et al., 2002; Greve and Fischl, 2009) and FMRIB's Nonlinear Image Registration Tool (FNIRT) by individual's high resolution T1-weighted structural image. The transformation matrix and the warp field from individual participants' diffusion space to the MNI standard space, and the inversed transformation matrix and warp field from the MNI standard space to individual diffusion space were obtained through the normalization process. The matrix and warp field were then used, respectively, for the striatum and nine target brain regions in the MNI standard space warping into participants' diffusion space, and for the normalization of the resulting tractography maps (scalar) into the MNI standard space. Visual inspection of normalized non-diffusion-weighted brain images was done to confirm that the registration was successful.

### Seed brain region and target brain regions

One seed mask and nine target masks, including the striatum, mOFC, lOFC, dlPFC, vlPFC, rostral CC, dorsal CC, PCC, hippocampus, and amygdala, were created for each hemisphere based on the automated anatomical labeling template (Tzourio-Mazoyer et al., 2002) and previous studies (Cohen et al., 2009; Lei et al., 2014). Detailed information of these masks was presented in our previous study (Lei et al., 2014). Figure S1 shows the anatomical locations of these brain masks for one hemisphere. All masks in the MNI standard space were transformed to individual diffusion space by using transformation matrix and warp field produced in the previous step and binarized. Volumes of these ten masks in individuals' diffusion space were obtained. The total intracranial volume (ICV) was obtained from high resolution T1-weighted anatomical image by using the Freesurfer segmentation software package (http://surfer.nmr.mgh.harvard.edu/; Fischl et al., 2002).

### Tractography and seed-based classification

Probabilistic tractography was performed from the striatum to the nine target regions in individuals' diffusion space by PROBTRACKS program implemented in FMRIB's diffusion toolbox (Behrens et al., 2003b). Five thousand tract-following samples were initiated in each voxel of the striatum, and were then tracked to the nine target regions, resulting in nine probabilistic maps of fiber connectivity, called tractography images. The value of each voxel in the tractography image represented the number of the tracking to the target region (connectivity between the voxel in the striatum to all voxels in the target region). Only voxels above the threshold of a minimum of 10 tracking samples per voxel were retained to assure true fiber connection after removing noise (Aron et al., 2007). The number of tracking samples of a given voxel to each target was then divided by the voxel's total tracking number to all regions to yield a proportional ratio. The resulting nine images were transformed back to the MNI standard space for group statistical analyses. The final spatial resolution was 1 mm^3^. All preprocessing was done separately for each hemisphere. Tractography images in the MNI standard space from two hemispheres were combined and used in general linear models to examine sex differences.

### Statistical analysis

Independent sample *t*-test in SPSS was used to examine sex differences in age, handedness, and ICV. Linear regression model with sex as a predictor and ICV as a covariate was used to examine sex differences in volumes of the ten brain regions.

Fiber connections between the striatum and nine target regions were analyzed using the general linear model with the tool “randomize” (Winkler et al., 2014). The permutation-based non-parametric test with the Threshold-Free Cluster Enhancement (TFCE) was used to perform group analysis (Nichols and Holmes, 2002; Smith and Nichols, 2009). This method has been highly recommended by the FSL group for research on finding statistically significant cluster-like structures. Nine preprocessed tractography images, each representing fiber connectivity between the striatum and one target region, were entered into nine general linear models, respectively. In order to exclude its potential confounding effect, the total intracranial volume (ICV) was entered into GLM as a covariate. Results were corrected for multiple comparisons with the TFCE algorithm (Smith and Nichols, 2009). We further extracted and plotted individual participants' average fiber connection within the striatal regions that showed significant sex differences.

## Results

**Figure 1:**
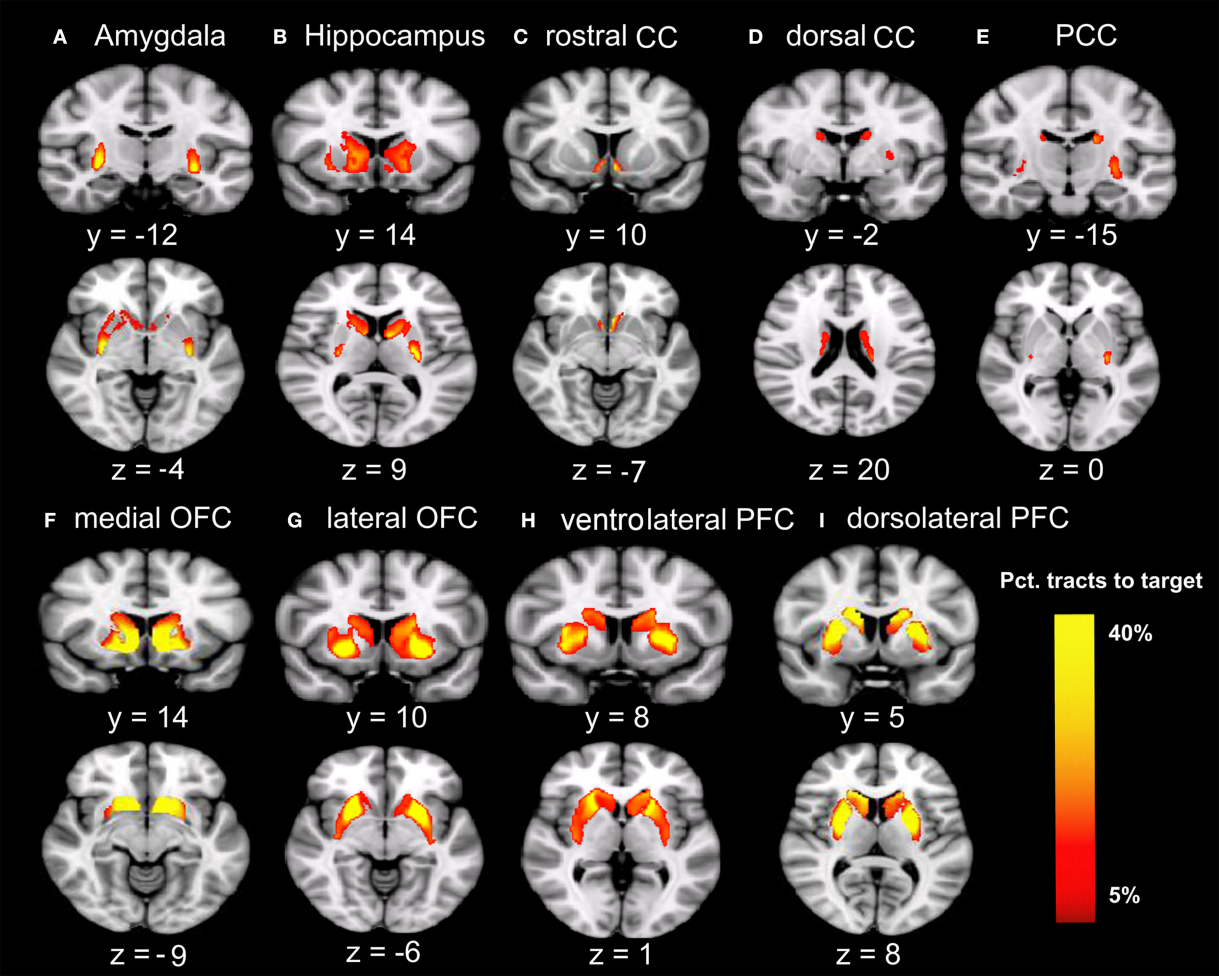
**Tracts between the striatum and each of the nine target regions**. Only voxels with at least 5% target-ending tracts are displayed. Colors indicate the proportions of target-specific tracts out of all tracts for a given voxel. mOFC, the medial orbitofrontal cortex; lOFC, the lateral orbitofrontal cortex; vlPFC, the ventrolateral prefrontal cortex; dlPFC, the dorsolateral prefrontal cortex; PCC, the posterior cingulate cortex/retrosplenial cortex; dorsal CC, the dorsal cingulate cortex; rostral CC, the rostral cingulate cortex; Amy, amygdala; Hipp, hippocampus. **(A)** Group-average tractography from the striatum to amygdala. **(B)** Group-average tractogragy from the striatum to hippocampus. **(C)** Group-average tractography from the striatum to rostral CC. **(D)** Group-average tractography from the striatum to dorsal CC. **(E)** Group-average tractography from the striatum to PCC. **(F)** Group-average tractography from the striatum to medial OFC. **(G)** Group-average tractography from the striatum to lateral OFC. **(H)** Group-average tractography from the striatum to ventrolatgeral PFC. **(I)** Group-average tractography from the striatum to dorsolateral PFC.

Males were found to have larger ICV and larger volumes in all ten brain regions included in this study (Table 1). Figure 1 shows the group–averaged tractography map of the connections between the striatum (including the putamen and caudate) and nine target regions. Clear anterior-posterior, medial-lateral, and dorsal-ventral connectivity patterns were observed. The rostral CC, mOFC, and lOFC were found to mainly connect to the medial and ventral parts of the striatum; the dlPFC, vlPFC, and dorsal CC to the dorsal and lateral parts of the striatum; the amygdala and hippocampus to the posterior putamen; and the PCC to the posterior caudate.

**Table 1:** **Descriptive statistics and sex differences**.

**Variables**	**All (*****n*** = **129)**		**Male (*****n*** = **50)**		**Female (*****n*** = **79)**		**Statistics**	
	**Mean**	***SD***	**Mean**	***SD***	**Mean**	***SD***	***T*^c^**	***p***
Age	20.36	0.84	20.52	0.84	20.25	0.83	−1.78	0.08
Handedness^a^	90.99	6.02	90.28	6.32	91.44	5.85	−0.1.07	0.29
**VOLUMES OF BRAIN REGIONS**^b^								
dlPFC	101601.8	11576.5	107762.7	11174.7	97702.5	10086.9	−4.71	<0.0001
vlPFC	28315.1	4851.6	30336.4	4809.9	27035.8	4451.5	−3.41	0.001
lOFC	32133.7	3217.6	33755.7	3277.2	31107.2	2736.5	−4.51	<0.0001
mOFC	30932.9	3566.8	33020.1	3100.7	29611.9	3205.8	−5.27	<0.0001
Rostral CC	16046.1	2607.6	17262.9	2613.4	15276.0	2306.0	−3.74	<0.0001
Dorsal CC	25764.2	2483.0	27156.9	2375.5	24882.7	2131.2	−5.03	<0.0001
PCC	42031.1	4901.4	44869.1	4624.3	40234.9	4190.3	−5.08	<0.0001
Amygdala	2841.3	371.3	3042.7	369.2	2713.9	313.3	−5.09	<0.0001
Hippocampus	11770.0	896.6	12343.5	929.0	11407.0	657.8	−6.03	<0.0001
Striatum	23843.3	2325.4	25192.8	1930.8	22989.2	2150.8	−5.04	<0.0001
ICV	1459216.8	231556.24	1543662.4	207768.4	1405770.2	231098.4	−3.43	0.001

a *Determined using Edinburgh Inventory (Oldfield, 1971); Scores greater than 0 indicate right-handedness. A score of 100 indicates strong right-handedness*.

b *Sex differences of the ten brain ROIs were analyzed with regression models with sex and ICV as predictors*.

c *Two kinds of T statistics are shown: independent sample T statistics for age, handedness, and ICV; and T statistics for the regression coefficients of sex as a predictor in regression*.

*ICV, intracranial volume; dlPFC, the dorsolateral prefrontal cortex; vlPFC, the ventrolateral prefrontal cortex; mOFC, the medial orbitofrontal cortex; lOFC, the lateral orbitofrontal cortex; rostral CC, the rostral cingulate cortex; dorsal CC, the dorsal cingulate cortex; PCC, the posterior cingulate cortex/retrosplenial cortex*.

**Figure 2:**
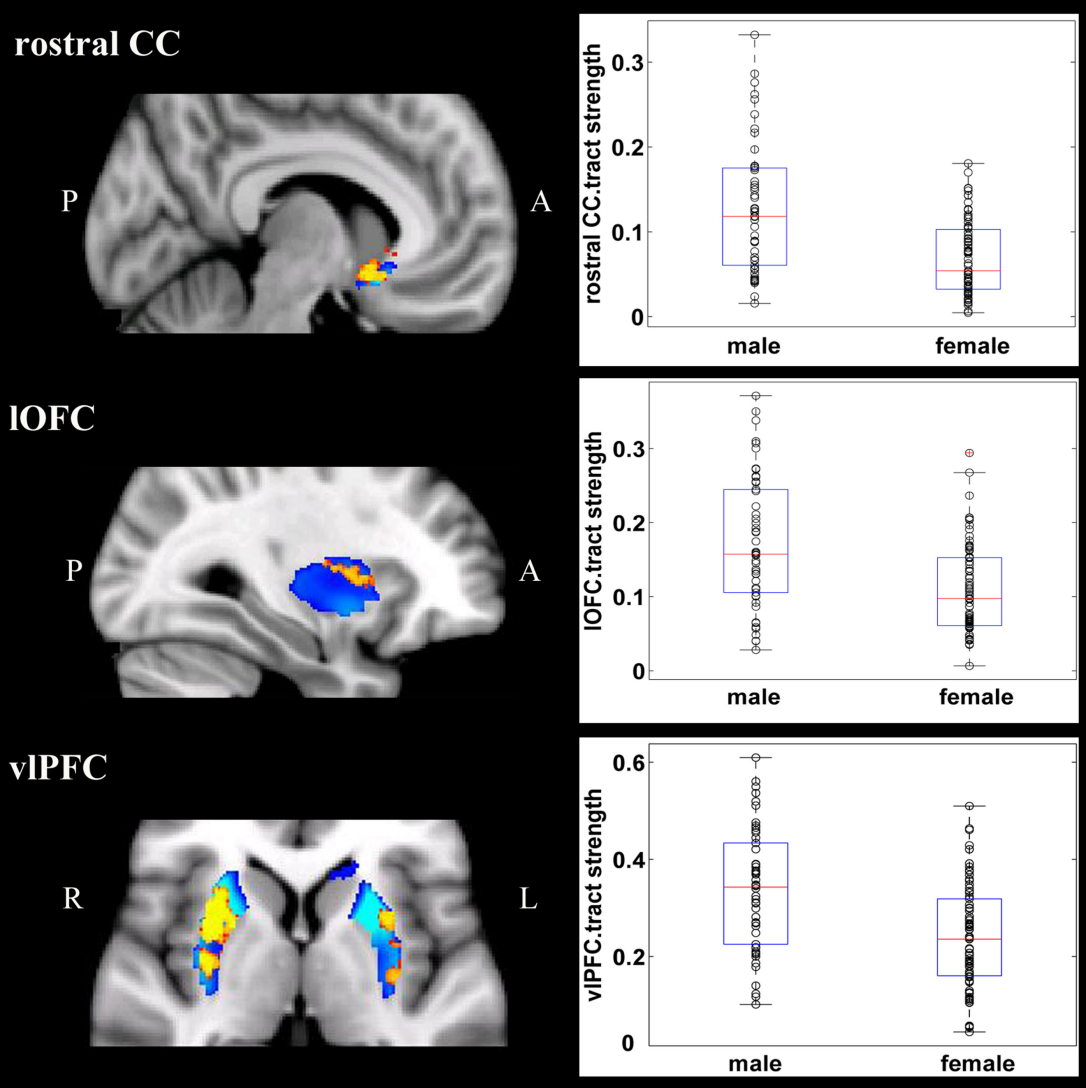
**Stronger fiber connection in males than in females**. Fiber connections between left caudate and rostral CC (top), between the right putamen and the lOFC (middle), between bilateral caudate and the vlPFC (bottom), and between the putamen and the vlPFC (bottom). Red, stronger connection for males than for females; Blue, proportion of target-specific tracts from Figure 1. See Figure 1 for abbreviations.

**Figure 3:**
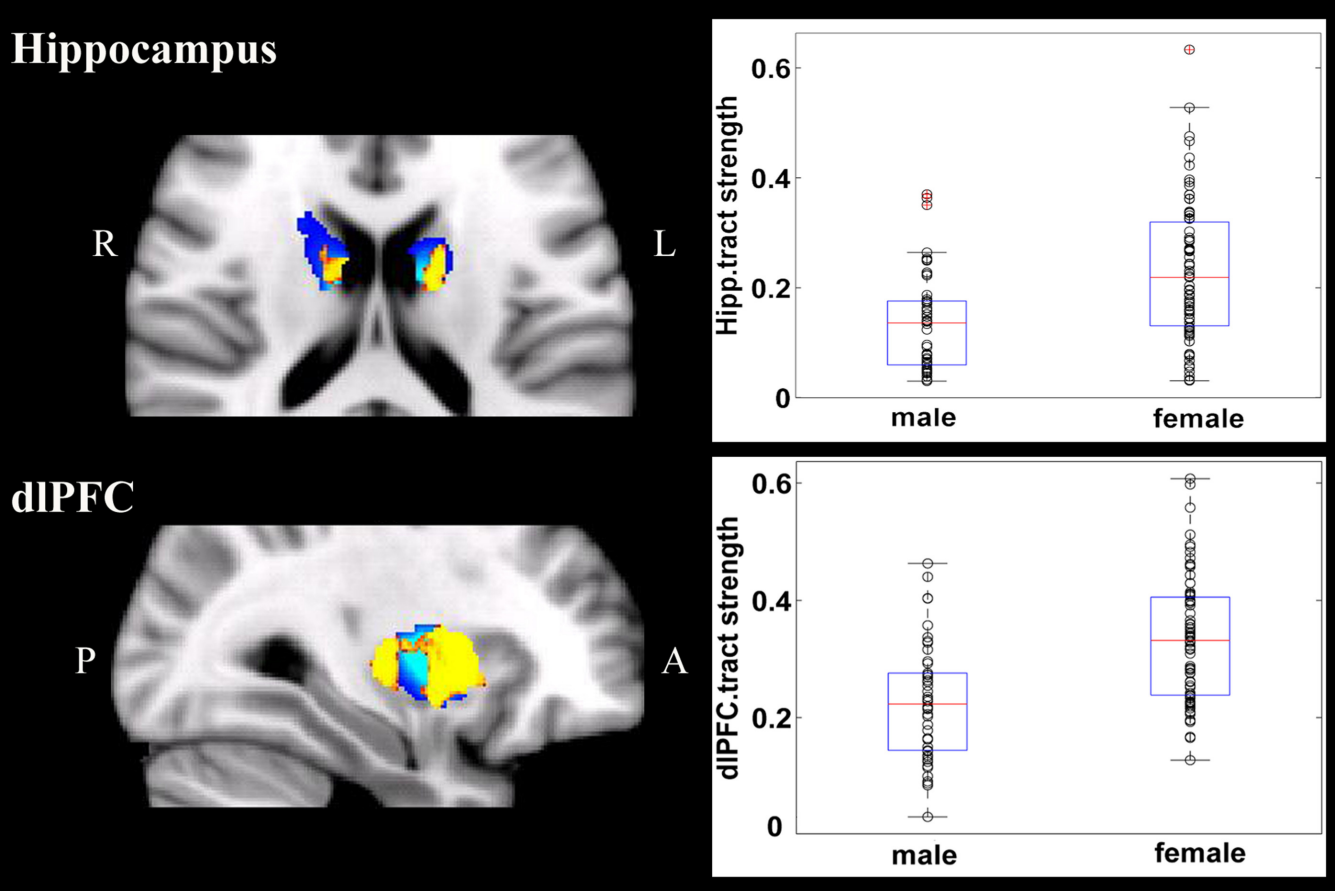
**Stronger fiber connection in females than in males**. Fiber connections between right putamen and the dlPFC (top), between the bilateral caudate and the hippocampus (bottom), and between the left putamen to the hippocampus (bottom). Yellow, stronger connection for females than for males; Blue, proportion of target-specific tracts from Figure 1. See Figure 1 for abbreviations.

Overall, females and males showed similar patterns of fiber connection (Figures S2, S3). General linear model analysis nevertheless revealed significant sex differences in fiber connection between sub-regions of the striatum and five of the nine target regions (the hippocampus, rostral CC, lOFC, vlPFC, and dlPFC). As shown in Table 2; Figures 2, 3, males showed greater fiber connection than did females between left ventral and medial caudate and the rostral CC, between the right ventrolateral putamen and the lOFC, between bilateral lateral putamen and the vlPFC, and between the left lateral caudate and the vlPFC, whereas females showed greater fiber connection between the right putamen and the hippocampus, between bilateral posterior caudate and the hippocampus, and between the right dorsolateral putamen and the dlPFC. These sex differences remained significant after controlling for the volumes of the striatum and the target region and ICV (see Table S1).

**Table 2:** **Fiber connectivity that showed significant sex differences**.

**Fiber target regions**	**Fiber-initiated regions in striatum**	**Volume in striatum/mm^3^**	**Tmax**	**MNI coordinate**
**MALES > FEMALES**				
Rostral CC	Left caudate	755	5.307	−4, 13, −5
lOFC	Right putamen	635	5.179	34, 3, 3
vlPFC	Right putamen	3578	4.576	33, 4, −5
	Left putamen	1130	4.218	−32, 0, −7
	Right caudate	192	4.601	13, 12, 20
	Right caudate	149	3.729	19, 6, 25
**MALES < FEMALES**				
dlPFC	Right putamen	3667	3.949	31, 0, −8
Hippocampus	Left putamen	840	3.653	−30, −12, −1
	Right caudate	693	3.801	11, −1, 15
	Left caudate	570	4.368	−14, −4, 17

*Rostral CC, the rostral cingulate cortex; lOFC, the lateral orbitofrontal cortex; vlPFC, the ventrolateral prefrontal cortex; dlPFC, the dorsolateral prefrontal cortex*.

## Discussion

The current study explored sex differences in fiber connectivity between the striatum and nine target regions. Consistent with previous studies (Gur et al., 1999; Goldstein et al., 2001; Cosgrove et al., 2007), we found larger ICV and brain regions in males than females. Group–averaged tractography patterns of the striatum were also consistent with the results of previous studies in primates and humans (Lehericy et al., 2004; Haber et al., 2006; Draganski et al., 2008; Haber and Knutson, 2010), which confirmed the validity of the current tractography. Despite overall similarities between males and females, significant sex differences in striatal fiber connectivity were observed in the current study. Males showed stronger fiber connection between the left ventromedial caudate and rostral CC, between the right ventrolateral putamen and the lOFC, between bilateral putamen and the vlPFC, and between the right lateral caudate and the vlPFC, whereas females showed stronger fiber connection between the right dorsolateral putamen and the dlPFC, between the bilateral posterior caudate and the hippocampus, and between the left lateral putamen and the hippocampus.

Several lines of previous research had hinted at potential sex differences in the striatum-connected fiber sub-networks found in this study. First, TBSS studies found that, compared to females, males had higher white matter integrity in white matter regions and tracts that underlie striatal-frontal connection (Wakana et al., 2004; Lawes et al., 2008; Nowinski et al., 2012), such as bilateral superior corona radiate (Takao et al., 2014), bilateral internal capsule, and cingulate (Hsu et al., 2008). Second, functional coupling between the striatum and anterior cingulate cortex and prefrontal cortex has been found to be stronger in males than in females in reward-related processing and impulse inhibition (Lighthall et al., 2012; Fattore, 2015), suggesting potentially stronger fiber connection in males. As reviewed in the Introduction, males were found to be more likely to recruit striatal-frontal circuits for reward-related processes and impulse inhibition (Diekhof et al., 2012; Lighthall et al., 2012). Consistently, several studies implicated striatal-cingulate and striatal-prefrontal circuits for reward, emotion and cognitive control (Beckmann et al., 2009; Dixon and Christoff, 2014; Burton et al., 2015; Dixon, 2015; Jarbo and Verstynen, 2015; Porter et al., 2015; Morris et al., 2016).

In contrast to the stronger fiber connection between right putamen to the vlPFC in males, females showed stronger fiber connection between right putamen and the dlPFC. Both vlPFC and dlPFC are central for reward-related cognitive control (Dixon and Christoff, 2014; Dixon, 2015). The vlPFC has been found to represent associations between rules and outcomes that are signaled by the immediate environment, and the dlPFC has been found to represent the relationship between more complex/abstract rules for action and desired outcomes, and to play a critical role in pursuing future reward (Dixon and Christoff, 2014; Dixon, 2015). Thus, this sex difference within striatal-lateral prefrontal circuits may reflect sex-specific neuroanatomical correlates of reward-related cognitive control.

Females also showed stronger fiber connections between the putamen and the hippocampus and between the caudate to the hippocampus than did males. In animal studies and human brain imaging studies, the striatum-hippocampus balance has been implicated in compensation and competition between different memory and learning systems (e.g., the hippocampus system for declarative learning and memory, and the striatum system for procedural learning and memory; Ghiglieri et al., 2011). Because functional coupling between the striatum and the hippocampus is involved in episodic memory (Jiang et al., 2015), our finding of stronger fiber connection between the striatum and hippocampus in females than in males may provide a structural neural basis for the robust sex differences in episodic memory (for meta-analyses, see Wang, 2013; Hyde, 2014). Future studies should specifically examine this speculation.

The current study had several limitations that need to be discussed. First, we only used probabilistic tractography, which does not allow for the identification of afferent or efferent striatal pathway (Rushworth et al., 2009; Bohanna et al., 2011). Second, our participants were young college students with a narrow age range, so we could not examine age-related changes in the striatal circuitry. Third, we found significant sex differences in the striatum-projected fiber connections that may explain behavioral differences, but how these brain differences came about remains unknown. Some researchers (Herting et al., 2012; Lentini et al., 2012) have discussed biological factors (e.g., sex hormones) involved in sex differences in brain anatomy, whereas others (Joel, 2011; Fine et al., 2013; Miller and Halpern, 2014; Rippon et al., 2014) have speculated about the importance of social environments. Fourth, based on previous research, we speculated that our finding of sex differences in the striatum-projected connection could account for some sex differences in behavior, but this study did not directly test that possibility. The prefrontal neurons have been found to be significantly more spinous than those in the other lobes, indicating a higher ability of prefrontal neurons to integrate a large number of excitatory inputs (Elston, 2000; Jacobs et al., 2001). Although the current study found that anatomical striatal-prefrontal circuitries differ in male and female, it should be noted that functional stimulations within these striatal-prefrontal circuitries may not have the same patterns of sex differences. The relationships between brain structure and function, as well between brain and behaviors, are complex, so great caution is needed when linking significant sex differences in striatal-prefrontal fiber connectivity to sex differences in behaviors.

## Conclusion

Using probabilistic tracking of diffusion tensor images, the current study found several significant sex differences in the fiber connection between the striatum and nine target regions after controlling for the ICV and the volumes of the striatum and target regions. These differences may help explain sex differences in relevant behaviors such as risky decision making, impulse inhibition, and memory.

## Author contributions

CC, GX, and QD designed this research. XL and LB analyzed the data. XL, ZH, and CC wrote this paper.

### Conflict of interest statement

The authors declare that the research was conducted in the absence of any commercial or financial relationships that could be construed as a potential conflict of interest.

## Supplementary material

Figure S1 **Anatomical locations of the nine target regions in one hemisphere**. Amy, amygdala; Hipp, hippocampus; mOFC, the medial orbitofrontal cortex; rostral CC, the rostral cingulate cortex; dorsal CC, the dorsal cingulate cortex; PCC, the posterior cingulate cortex/retrosplenial cortex; lOFC, the lateral orbitofrontal cortex; vlPFC, the ventrolateral prefrontal cortex; dlPFC, the dorsolateral prefrontal cortex.

Figure S2 **Tracts between the striatum and each target region for males**. Only voxels with at least 5% target-ending tracts are displayed. Colors indicate proportion of target-specific tracts out of all tracts for a given voxel. See Figure S1 for abbreviations.

Figure S3 **Tracts between the striatum and each target region for females**. Only voxels with at least 5% target-ending tracts are displayed. Colors indicate proportion of target-specific tracts out of all tracts for a given voxel. See Figure S1 for abbreviations.

Table S1 **Linear regression models for the nine fiber connections from Table 2**.

## Abbreviations

DTI: diffusion tensor imaging
mOFC: the medial orbitofrontal cortex
lOFC: the lateral orbitofrontal cortex
vlPFC: the ventrolateral prefrontal cortex
dlPFC: the dorsolateral prefrontal cortex
PCC: the posterior cingulate cortex/retrosplenial cortex
dorsal CC: the dorsal cingulate cortex
rostral CC: the rostral cingulate cortex
Amy: amygdala
Hipp: hippocampus.
