# Supplementary figures and images for: Sex Differences in Fiber Connection between the Striatum and Subcortical and Cortical Regions
Source: Front Comput Neurosci. 2016 Sep 23;10:100. doi: 10.3389/fncom.2016.00100 (PMC5034007; doi:10.3389/fncom.2016.00100)

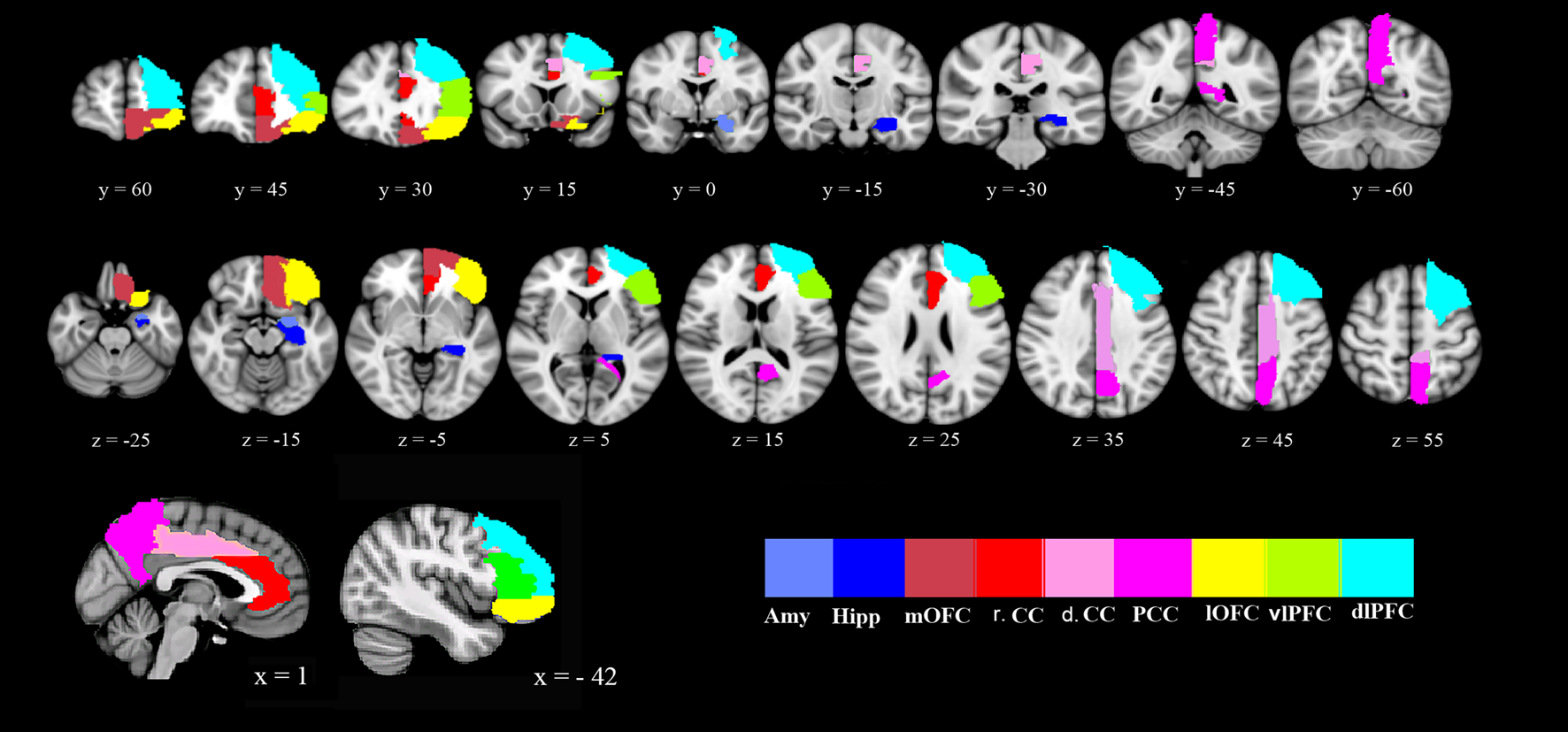

Supplement: Figure S1 — Anatomical locations of the nine target regions in one hemisphere. Amy, amygdala; Hipp, hippocampus; mOFC, the medial orbitofrontal cortex; rostral CC, the rostral cingulate cortex; dorsal CC, the dorsal cingulate cortex; PCC, the posterior cingulate cortex/retrosplenial cortex; lOFC, the lateral orbitofrontal cortex; vlPFC, the ventrolateral prefrontal cortex; dlPFC, the dorsolateral prefrontal cortex. [file Image1.TIF]

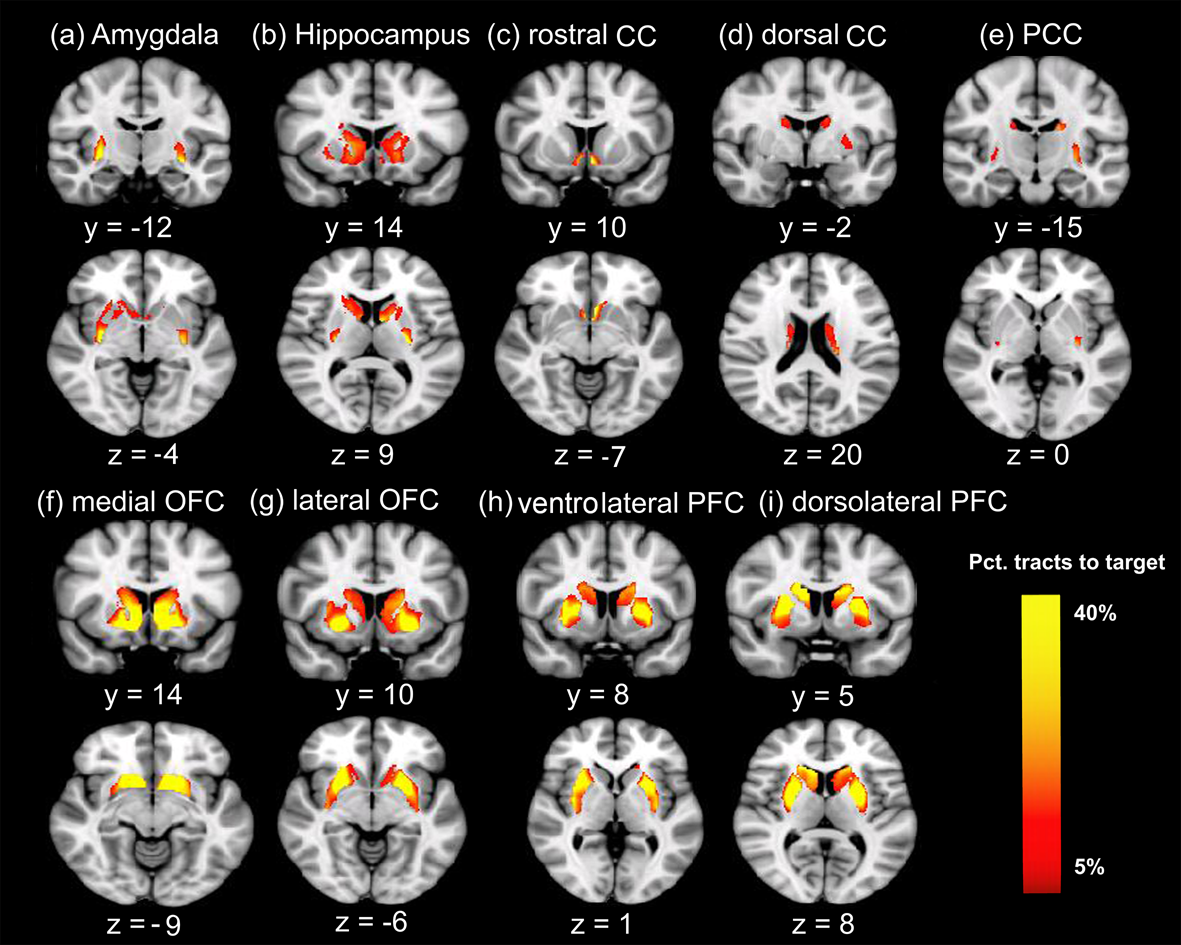

Supplement: Figure S2 — Tracts between the striatum and each target region for males. Only voxels with at least 5% target-ending tracts are displayed. Colors indicate proportion of target-specific tracts out of all tracts for a given voxel. See Figure S1 for abbreviations. [file Image2.TIF]

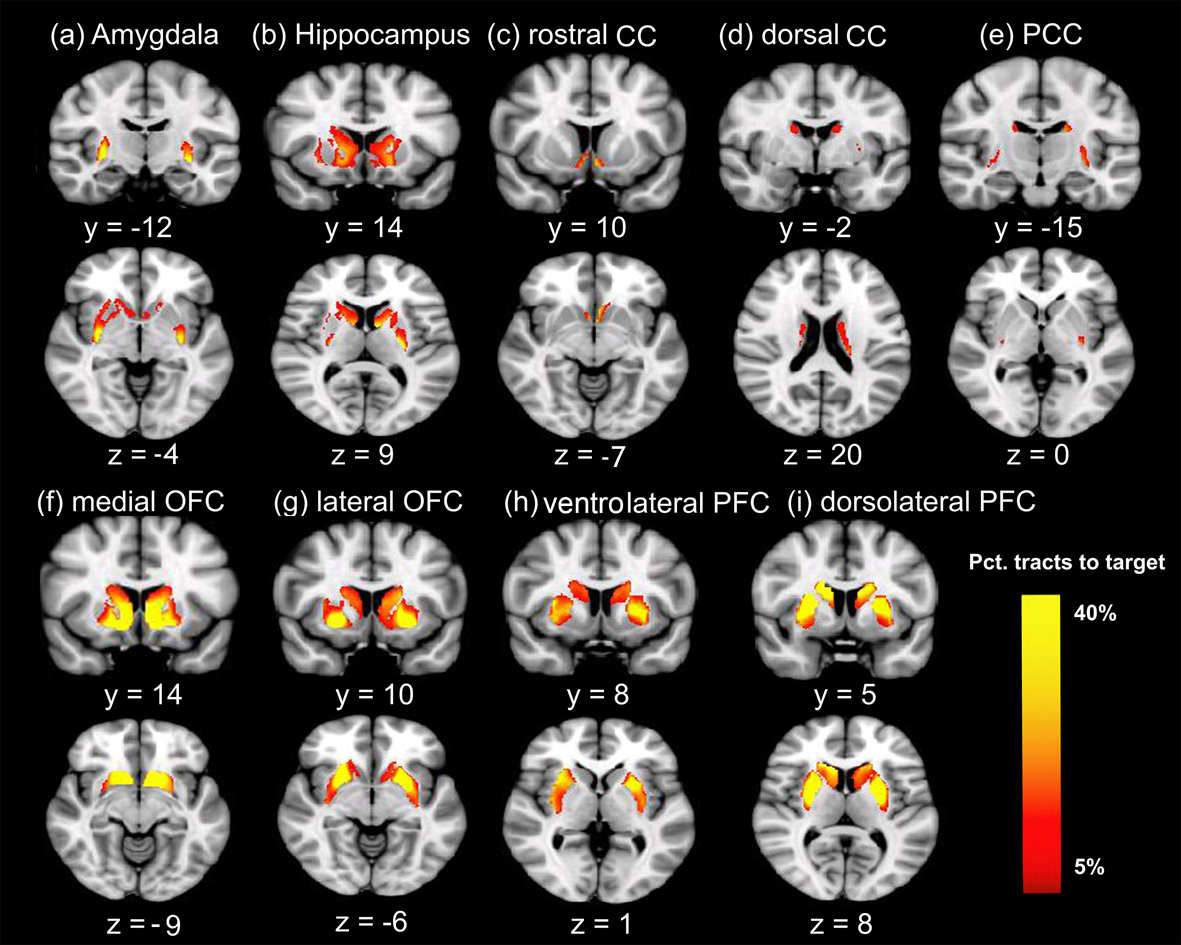

Supplement: Figure S3 — Tracts between the striatum and each target region for females. Only voxels with at least 5% target-ending tracts are displayed. Colors indicate proportion of target-specific tracts out of all tracts for a given voxel. See Figure S1 for abbreviations. [file Image3.TIF]
