# Supplementary material for: Sex Differences in Fiber Connection between the Striatum and Subcortical and Cortical Regions
Source: Front Comput Neurosci. 2016 Sep 23;10:100. doi: 10.3389/fncom.2016.00100 (PMC5034007; doi:10.3389/fncom.2016.00100)
Supplement: Table S1 — Linear regression models for the nine fiber connections from Table 2. [file Table1.docx]

Table S1 Linear regression models for the nine fiber connections from Table 2.

| Fiber connection | | Beta | T | *P* |
| --- | --- | --- | --- | --- |
| **Male > female** | |  |  |  |
| Between left caudate and rostral CC | | F (4,128)=7.464, *p* = 0.000020, adjusted *R*^2^=0.168 | | |
|  | ICV | -.123 | -1.422 | .158 |
|  | Striatum volume | -.009 | -.090 | .928 |
|  | Rostral CC volume | -.025 | -.260 | .795 |
|  | Sex | -.471 | -5.041 | **.000** |
| Between right putamen and lOFC | | F (4,128)=8.791, *p* = 0.000003, adjusted *R*^2^=0.196 | | |
|  | ICV | .001 | .009 | .993 |
|  | Striatum volume | -.248 | -2.591 | **.011** |
|  | lOFC volume | .032 | .353 | .725 |
|  | Sex | -.521 | -5.552 | **.000** |
| Between right putamen and vlPFC | | F (4,128)=9.880,*p* = 5.6803E-7, adjusted *R*^2^=0.217 | | |
|  | ICV | -.024 | -.289 | .773 |
|  | Striatum volume | -.266 | -2.741 | **.007** |
|  | vlPFC volume | .166 | 1.848 | .067 |
|  | Sex | -.509 | -5.624 | **.000** |
| Between left putamen and vlPFC | | F (4,128)=6.750, *p* = 0.000060, adjusted *R*^2^=0.152 | | |
|  | ICV | .013 | .146 | .884 |
|  | Striatum volume | -.142 | -1.406 | .162 |
|  | vlPFC volume | .162 | 1.728 | .086 |
|  | Sex | -.401 | -4.265 | **.000** |
| Between right caudate and vlPFC | | F (4,128)=7.073, *p* = 0.000037, adjusted *R*^2^=0.16 | | |
|  | ICV | -.019 | -.221 | .825 |
|  | Striatum volume | -.108 | -1.078 | .283 |
|  | vlPFC | .248 | 2.658 | **.009** |
|  | Sex | -.347 | -3.698 | **.000** |
| **Female > male** | | | | |
| Between right putamen and dlPFC | | F (4,128)=12.10, *p* = 2.466E-8, adjusted *R*^2^=0.258 | | |
|  | ICV | .131 | 1.614 | .109 |
|  | Striatum volume | .297 | 2.836 | **.005** |
|  | dlPFC volume | -.055 | -.549 | .584 |
|  | Sex | .594 | 6.690 | **.000** |
| Between left putamen and hippocampus | | F (4,128)=7.188, *p* = 0.000031, adjusted *R*^2^=0.162 | | |
|  | ICV | -.037 | -.433 | .666 |
|  | Striatum volume | -.046 | -.442 | .659 |
|  | Hippocampus volume | .099 | .936 | .351 |
|  | Sex | .444 | 4.529 | **.000** |
| Between right caudate and hippocampus | | F (4,128)=6.425, *p* = 0.000099, adjusted *R*^2^=0.145 | | |
|  | ICV | .113 | 1.292 | .199 |
|  | Striatum volume | .143 | 1.359 | .177 |
|  | Hippocampus volume | -.172 | -1.615 | .109 |
|  | Sex | .381 | 3.850 | **.000** |
| Between left caudate and hippocampus | | F (4,128)=5.410, *p*=0.000476, adjusted *R*^2^=0.121 | | |
|  | ICV | .046 | .514 | .608 |
|  | Striatum volume | -.013 | -.119 | .906 |
|  | Hippocampus volume | -.062 | -.575 | .566 |
|  | Sex | .355 | 3.534 | **.001** |

Abbreviations: rostral CC, the rostral cingulate cortex; lOFC, the lateral orbitofrontal cortex; vlPFC, the ventrolateral prefrontal cortex; dlPFC, the dorsolateral prefrontal cortex.
